# Status Quo and Research Trends of Craniopharyngioma Research: A 10-Year Bibliometric Analyses (From 2011 to 2020)

**DOI:** 10.3389/fonc.2021.744308

**Published:** 2021-09-30

**Authors:** Tianhua Li, Anming Yang, Guangjie Liu, Shisheng Zou, Yiguang Chen, Bowen Ni, Yi Liu, Jun Fan

**Affiliations:** ^1^ Department of Neurosurgery, Nanfang Hospital, Southern Medical University, Guangzhou, China; ^2^ The Laboratory for Precision Neurosurgery, Nanfang Hospital, Southern Medical University, Guangzhou, China

**Keywords:** craniopharyngioma, bibliometrics, citation, hotspots, trends

## Abstract

**Background:**

Craniopharyngioma (CP) is a challenging intracranial tumor due to its special hypothalamus-pituitary location. Each patient with CP should be evaluated and treated separately. Exploring novel methods of automatized analysis of data for gaining knowledge on any medical field is an encouraging task, particularly in such an extremely challenging tumor as CP. We aim to summary the situations, investigate the research trends and evaluate research hotspots using bibliometric analysis for the CP research.

**Methods:**

We extracted all the CP-related literatures from 2011 to 2020 from the Web of Science database. An Online analysis platform of literature metrology (Bibliometric), BICOMB, gCLUTO and CiteSpace softwares were used to do bibliometric analysis. As a supplement, we also analyzed the top 100 cited case reports with particular and certainly infrequent information to improve the analysis.

**Results:**

According to our retrieval strategy, we found a total of 1262 CP-related literatures. The United States has maintained a leading position in global CP research, followed by China and Germany. Among institutions, Capital Med Univ, St Jude Childrens Res Hosp and Southern Med Univ rank in the top 3 in terms of the number of articles published. “WORLD NEUROSURGERY” is the most popular journal for CP-related research. Moreover, MULLER HL, MERCHANT TE, QI ST and others have made great achievements in the study of CP. Finally, we did biclustering analysis on keywords and identified 4 CP research hotspot clusters.

**Conclusions:**

Our research provides a comprehensive analysis of the scientific progress of CP in the past 10 years, and insight into the development of CP research field, highlight research trends over time, and help identify valuable future directions.

## Introduction

Craniopharyngioma (CP) is an epithelial tumor that origins from remnants of the craniopharyngeal duct epithelium. There are 0.5-2.5 new cases per million people per year worldwide. They account for 1.2-4.6% of all intracranial tumors and 5-11% of brain tumors in children (1–3). In children, symptoms such as central diabetes insipidus, visual impairment, headache, and growth retardation are highly indicative of CP. In adults, increased intracranial pressure (such as headache), endocrine defects (such as impaired sexual function), and hypothalamic syndrome (such as disturbances in water balance and body temperature regulation) are the main symptoms. CP includes adamantinomatous craniopharyngioma (ACP) and papillary craniopharyngioma (PCP). ACP is a more common subtype that affects people of all ages, while PCP is mostly limited to adults (4).

Different from other medical fields, CP is one of the most complex tumors could be analyzed by automatic algorithm. Although CP is a pathologically benign tumor and its histological grade is WHO I, it often affects the prognosis and outcome of patients because CP occurs in the special location of the hypothalamus-pituitary axis (1). There is no fixed treatment plan that is effective for this kind of pathology. Therefore, each patient with CP should be evaluated and treated separately.

With the continuous improvement of the quality of life and medicine, people pay more attention to CP. However, the large number of variables involved in the results of each patient and the expertise gained from surgical treatment vary greatly among authors, which brings greater complexity to the analysis of results. As early as the 1930s, Harvey Cushing, the first neurosurgeon to deal with these lesions extensively, predicted this problem (2).

It is an encouraging task to explore new methods for automated data analysis to acquire knowledge in any medical field, especially in such an extremely challenging tumor as CP. Through bibliometric methods and tools, we can make a more macro analysis on the basis of a large amount of literature data, which has become an important method to grasp the development trend of a field accurately. However, there are very few bibliometric studies on CP. Only Gnacio Jusue-Torres et al. summarized the 100 most cited articles (3). This literature lacks an analysis of the relationship among countries/regions, institutions, and authors, and prediction of CP research hotspots. And only analyzed the most cited 100 literatures, the scope is not large enough. Previous studies have shown that biclustering analysis can help to identify critical areas of research and relevant representative literatures (4). In this paper, through a comprehensive analysis of the relevant literatures and external characteristics of CP, we summarized various information of articles about CP and help identify valuable future directions.

## Materials And Methods

### Data Search and Download

Based on the Science Citation Index-Expanded database, the bibliometric analysis was carried out. In the Web of Science database, we set the subject word (search title, abstract, author and keywords, etc.): craniopharyngioma; time: 2011-2020; article types: original articles and reviews. Through the above search strategy, the literatures related to CP were obtained. At the same time, in order to make our research more comprehensive, we included top 100 cited case reports during this period in the analysis, which could provide information on particular and certainly infrequent cases.

### Data Collection

The data including fully recorded and quoted references was downloaded from Web of Science and imported into the Online Analysis Platform of Bibliometrics (http://bibliometric.com/) and CiteSpace (version 5.7.R3, Drexel University, USA). CiteSpace is an outstanding tool for collaborative network analysis to connect various publication features. Import the data downloaded from Web of Science database into it, and click the relevant categories to analyze the relationship among countries, institutions, authors, etc. By obtaining keywords with high citation rate, the research frontier and emerging trends in this field are predicted (5). Bibliographic Item Co-Occurrence Matrix Builder (BICOMB, version 2.01, China Medical University, China) (6) and gCLUTO (version1.0, University of Minnesota, USA) software for bibliometric analysis and corresponding clustering can be obtained. BICOMB can generate a binary matrix with source literatures as columns and keywords as rows. Import the binary matrix into gCLUTO, select the default setting, and adjust the number of clusters to get the appropriate cluster.

### Bibliometric Analysis

We included publishing characteristics such as countries, institutions, journals, authors, and H-index in our analysis. At the same time, the 2020 version of Journal Citation Reports (JCR) and impact factor (IF), as important indicators to measure the scientific value of research, were also included in the analysis (7). Using the bibliometric, we analyzed the changes in the volume of articles and the cooperative relations of various countries. In CiteSpace, we connected all kinds of publication characteristics through collaborative network analysis. At the same time, we also obtained highly cited keywords to predict the research frontiers and emerging trends in this field (5, 6).

### Biclustering Analysis of Research Hotspots

Biclustering can be used to show the relationship between frequent keywords, as well as the relationship between frequent keywords and the original literatures. In order to investigate the research hotspots of CP, we carried out the biclustering analysis of the publications and keywords. Through BICOMB, we constructed a binary matrix with source documents as columns and keywords as rows. Then used the software gCLUTO to analyze the matrix (4, 8). Finally, the semantic relationship between keywords and source documents was drawn through mountain and matrix visualization.

## Results

### The Search Results of Related Literatures

**Figure 1:**
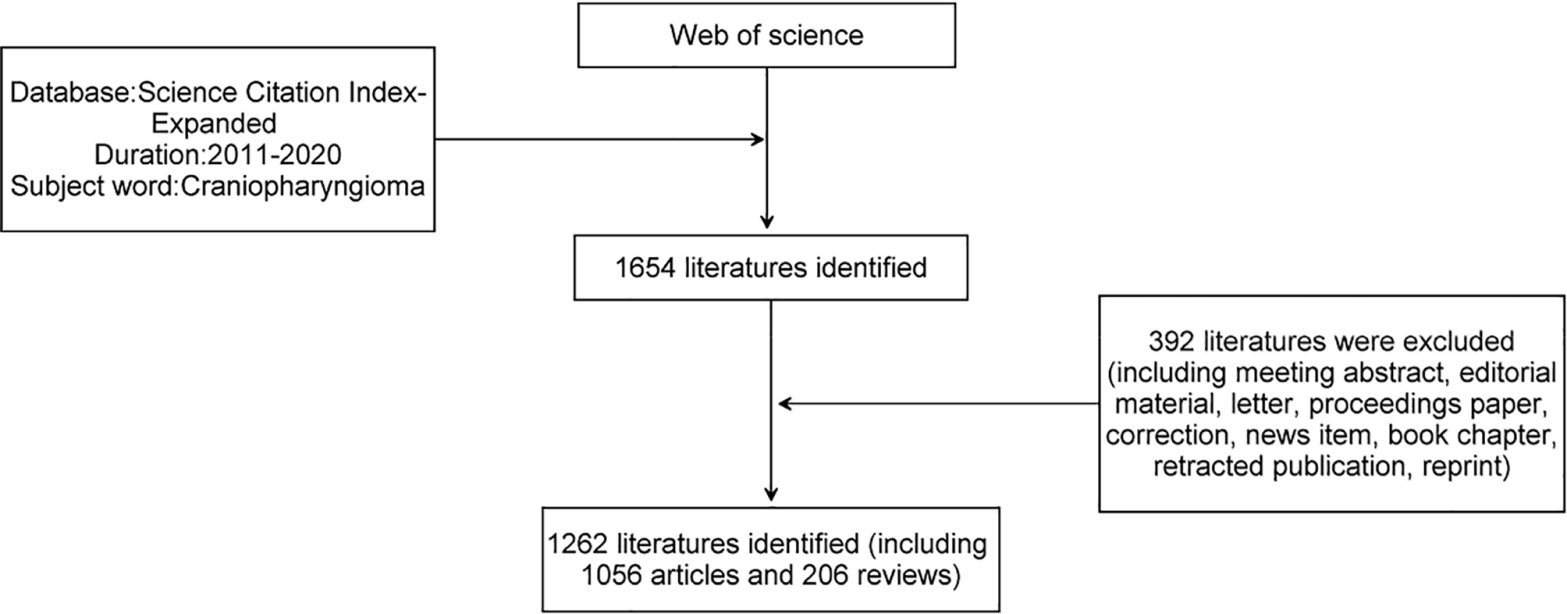
Flow chart of literature filtering.

The flow chart of the search is shown in **Figure 1**. According to our strategy, a total of 1262 related literatures were retrieved from 2011 to 2020.

### The Contributions of Countries and Institutions to Global Publications

**Figure 2:**
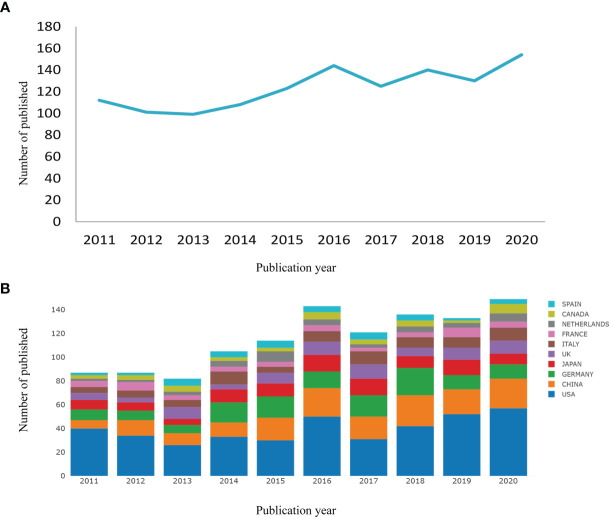
Output of related literature. The number of annual publications **(A)** and growth trends of the top 10 countries/regions **(B)** in craniopharyngioma research from 2011 to 2020. Conducted by online analysis platform of Bibliometrics.

The volume of articles related to CP in the past 10 years is shown in **Figure 2A**. Generally speaking, there is little fluctuation in the volume of articles. Among them, the number of literature is the largest in 2020 (154) and the lowest in 2013 (99). The number of literature in the past five years has increased significantly compared with the previous five years. At least 57 countries and regions are doing research on CP. In the past 10 years, the United States has made the most significant contribution to the study of CP, followed by China, Germany, Japan etc. (**Figure 2B**). Centrality can be used to measure the importance of nodes in the network. The higher the centrality, the greater the importance of nodes. The results showed that the influence of the United States is more significant than that of any other country (centrality = 0.33), followed by England (0.22) and Germany (0.20) (**Table 1**).

**Table 1:** The top 10 countries/regions and institutions contributing to publications in craniopharyngioma research.

Rank	Countries	Article counts	Percentage	Centrality	Institutions	Article counts	Centrality	Total number of citation	Average number of citations
1	USA	405	32.09%	0.33	Capital Med Univ	32	0.07	199	6.22
2	PEOPLES R CHINA	172	13.63%	0.01	St Jude Childrens Res Hosp	30	0.06	413	13.77
3	GERMANY	141	11.17%	0.2	Southern Med Univ	28	0.16	175	6.25
4	JAPAN	103	8.16%	0.02	Univ Calif San Francisco	24	0.05	558	23.25
5	ITALY	85	6.74%	0.15	Harvard Med Sch	23	0.05	109	4.74
6	ENGLAND	78	6.18%	0.22	UCL	23	0.22	477	20.74
7	FRANCE	53	4.20%	0.05	La Princesa Univ Hosp	22	0.01	416	18.91
8	NETHERLANDS	47	3.72%	0.01	Univ Florida	22	0.03	251	11.41
9	CANADA	44	3.49%	0.01	Sichuan Univ	20	0.02	89	4.45
10	SPAIN	44	3.49%	0.05	Univ Colorado	20	0.04	162	8.1

**Figure 3:**
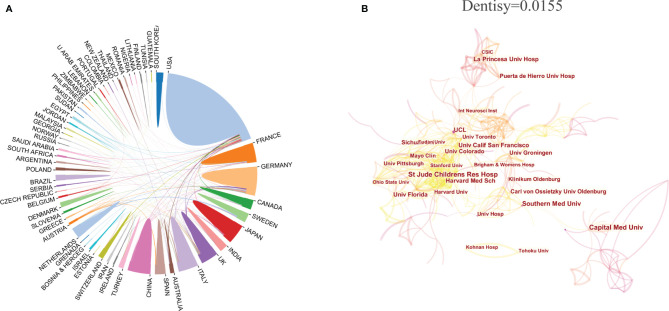
The distribution of countries/regions and institutions. Cooperative relations among countries/regions **(A)** (The area represents the number of articles, and the connection represents the cooperative relationship. Conducted by online analysis platform of Bibliometrics) and institutions **(B)**. [Conducted by CiteSpace (version 5.7.R3, Drexel University)].

The analysis of international cooperation shows that the United States cooperates frequently with other countries. Although China ranks second in the number of articles published, it cooperates less with other countries (**Figure 3A**). In terms of research institutions, the top 10 (**Table 1**) included Capital Med Univ(32), St Jude Childrens Res Hosp(30), Southern Med Univ(28), Univ Calif San Francisco(24), Harvard Med Sch(23),UCL(23), La Princesa Univ Hosp(22), Univ Florida(22), Sichuan Univ(20), Univ Colorado(20). The network density of CP research is only 0.0155 (**Figure 3B**), meaning that the research teams are relatively dispersed in several institutions and do not cooperate closely enough. Among these 10 institutions, only the centrality of Southern Med Univ(0.16) and UCL(0.22) is greater than 0.1, which indicates that the influence degree and cooperation degree of these two institutions are high in recent 10 years. However, on the whole, most institutions have a low degree of influence and lack of cooperation.

### Journals Publishing Researches on CP

In this 10 years, 313 journals publish literatures in the field of CP. Of the 1262 articles on CP we studied, the top 10 journals published 460 (44.43%) (**Table 2**). In terms of the number of publications, the top 3 are WORLD NEUROSURGERY (IF=1.829), CHILDS NERVOUS SYSTEM (IF=1.298) and JOURNAL OF NEUROSURGERY (IF=3.968). In terms of the average number of citations, the top 3 are JOURNAL OF CLINICAL ENDOCRINOLOGY (IF=5.399), EUROPEAN JOURNAL OF ENDOCRINOLOGY (IF=5.308) and JOURNAL OF NEUROSURGERY (IF=3.968). According to JCR of 2020, the 3 journals belong to Q1.

**Table 2:** The top 10 most active journals that published articles in craniopharyngioma research (sorted by count).

Rank	Journal title	Article counts	Percentage	Total number of citations	Average number of citations	IF	JCR	H-index
1	WORLD NEUROSURGERY	102	8.08%	904	8.863	2.103	Q3	16
2	CHILDS NERVOUS SYSTEM	60	4.75%	480	8.000	1.475	Q4	11
3	JOURNAL OF NEUROSURGERY	54	4.28%	1281	23.722	5.112	Q1	21
4	PITUITARY	48	3.80%	619	12.896	4.102	Q2	14
5	JOURNAL OF NEURO ONCOLOGY	39	3.09%	594	15.231	4.13	Q2	14
6	NEUROSURGICAL FOCUS	39	3.09%	688	17.641	4.044	Q1	17
7	JOURNAL OF NEUROSURGERY PEDIATRICS	31	2.46%	257	8.290	2.372	Q2	9
8	JOURNAL OF CLINICAL ENDOCRINOLOGY METABOLISM	30	2.38%	877	29.233	5.958	Q1	15
9	ACTA NEUROCHIRURGICA	29	2.30%	257	8.862	2.212	Q3	8
10	EUROPEAN JOURNAL OF ENDOCRINOLOGY	28	2.22%	675	24.107	6.661	Q1	17

### The Contributions of Authors to CP Research

**Figure 4:**
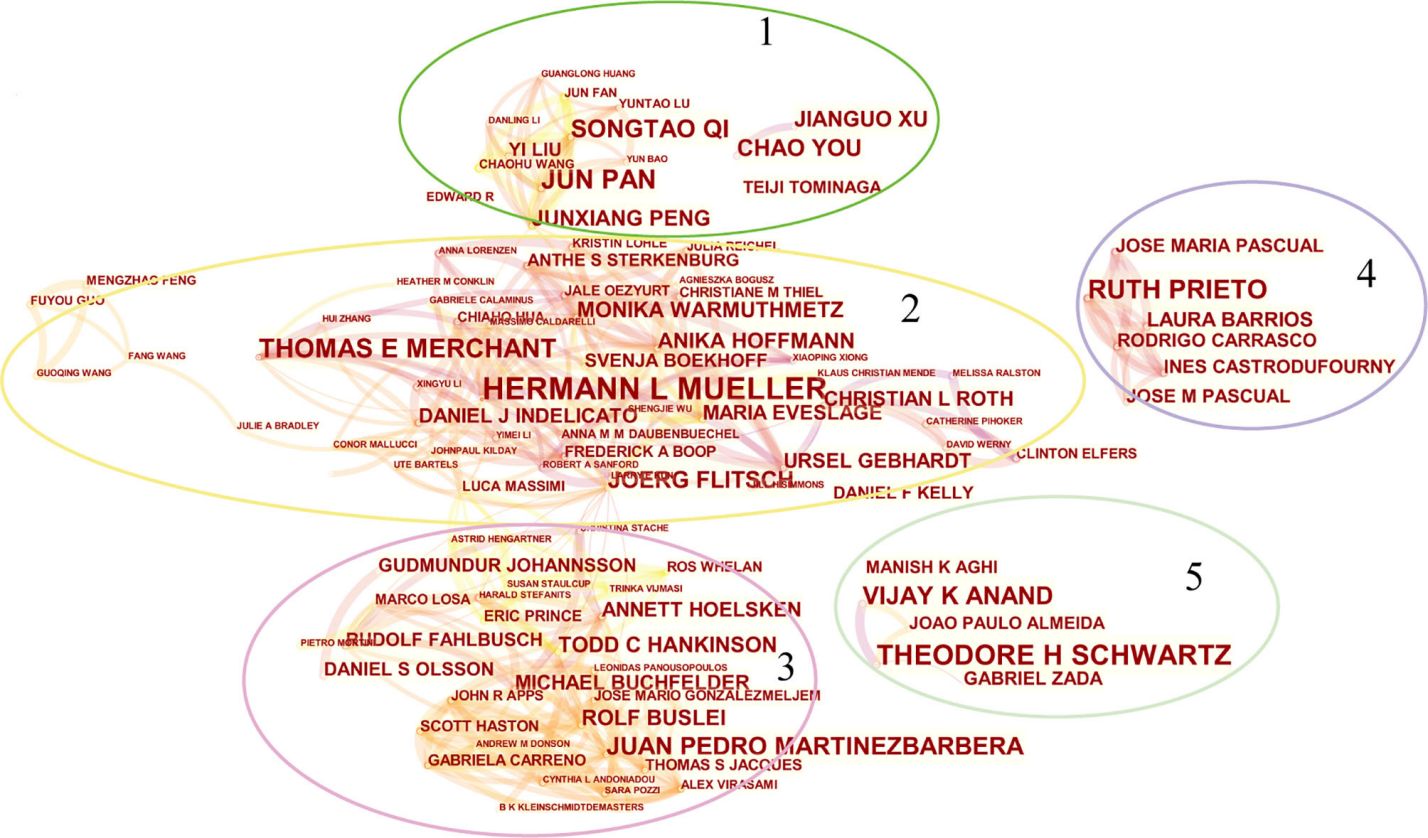
The network map of productive authors. Conducted by CiteSpace (version 5.7.R3, Drexel University).

The top 10 authors (the number of literature published) were listed in **Table 3**. Among those, HERMANN L MUELLER, from the Department of Pediatrics and Pediatric Hematology/Oncology, University Children’s Hospital, ranked first. At the same time, he is also at the first of the list in the analysis of co-cited authors. And 2 of the top 10 high-cited papers were published by him (**Table 4**). Those show that MUELLER has made outstanding achievements in CP researches. Through CiteSpace, we analyzed the author’s citation information and visualized it into a network (**Figure 4**). According to the clustering information, the top 10 authors were roughly divided into five modules. Module 1 includes QI ST, PAN J and XU JG. They are all from China, of which QI ST and PAN J are from the same unit, Southern Medical University, Nanfang Hospital, Department of Neurosurgery. Module 2 includes MULLER HL and MERCHANT TE. In recent 10 years, they occupy the top 2 in terms of the number of articles about CP. Simultaneously, the module they represent contains a large number of researchers and is closely related to other modules, occupying a core position in the study of CP. Module 3 contains many authors who have made outstanding contributions to CP, but only MARTINEZ-BARBERA JP is in the top 10. Module 4 includes PASCUAL JM and PRIETO R. Module 5 includes SCHWARTZ TH and ANAND VK. These two modules contain few authors and have little connection with other modules, but their contribution to CP is also outstanding.

**Table 3:** The top 10 most productive authors and co-cited authors contributed to publications in craniopharyngioma research.

Rank	Author	Article counts	Total citation	Average number of citations	H-index	Modules	Co-cited author	Citation counts
1	MULLER HL	51	1242	24.353	21	2	MULLER HL	378
2	MERCHANT TE	28	476	17.000	12	2	KARAVITAKI N	309
3	QI ST	27	203	7.519	9	1	FAHLBUSCH R	206
4	SCHWARTZ TH	27	723	26.778	13	5	YASARGIL MG	183
5	PAN J	25	187	7.480	9	1	BUNIN GR	169
6	PASCUAL JM	25	427	17.080	12	4	MERCHANT TE	161
7	PRIETO R	25	427	17.080	12	4	VAN EFFENTERRE R	155
8	ANAND VK	21	621	29.571	11	5	DEVILE CJ	155
9	MARTINEZ-BARBERA JP	21	594	28.286	14	3	PUGET S	154
10	XU JG	19	80	4.211	6	1	ELLIOTT RE	147

**Table 4:** The top 10 high-cited papers in craniopharyngioma research during 2011 to 2020.

Rank	Title	Journal	Authors	Publication year	Total citation	JCR	IF
1	Increased Wingless (Wnt) signaling in pituitary progenitor/stem cells gives rise to pituitary tumors in mice and humans	PROCEEDINGS OF THE NATIONAL ACADEMY OF SCIENCES OF THE UNITED STATES OF AMERICA	Gaston-Massuet	2011	153	Q1	11.201
2	Craniopharyngioma	ENDOCRINE REVIEWS	Mueller, Hermann L.	2014	137	Q1	19.874
3	Post-operative hypothalamic lesions and obesity in childhood craniopharyngioma: results of the multinational prospective trial KRANIOPHARYNGEOM 2000 after 3-year follow-up	EUROPEAN JOURNAL OF ENDOCRINOLOGY	Mueller, Hermann L.	2011	124	Q1	6.66
4	Endoscopic Endonasal Compared with Microscopic Transsphenoidal and Open Transcranial Resection of Craniopharyngiomas	WORLD NEUROSURGERY	Komotar, Ricardo J.	2012	120	Q3	2.103
5	Neurosurgical treatment of craniopharyngioma in adults and children: early and long-term results in a large case series	JOURNAL OF NEUROSURGERY	Mortini, Pietro	2011	119	Q1	5.11
6	Endoscopic, Endonasal Resection of Craniopharyngiomas: Analysis of Outcome Including Extent of Resection, Cerebrospinal Fluid Leak, Return to Preoperative Productivity, and Body Mass Index	NEUROSURGERY	Leng, Lewis Z.	2012	111	Q1	4.653
7	Endoscopic endonasal surgery for craniopharyngiomas: surgical outcome in 64 patients Clinical article	JOURNAL OF NEUROSURGERY	Koutourousiou	2013	109	Q1	5.111
8	Pituitary Magnetic Resonance Imaging for Sellar and Parasellar Masses: Ten-Year Experience in 2598 Patients	JOURNAL OF CLINICAL ENDOCRINOLOGY & METABOLISM	Famini	2011	108	Q1	5.958
9	Gasket Seal Closure for Extended Endonasal Endoscopic Skull Base Surgery: Efficacy in a Large Case Series	WORLD NEUROSURGERY	Garcia-Navarro	2013	103	Q3	2.103
10	The endoscopic endonasal approach for the management of craniopharyngiomas: a series of 103 patients	JOURNAL OF NEUROSURGERY	Cavallo, Luigi Maria	2014	102	Q1	5.111

### Analysis of the Top 100 Case Reports

We searched and retrieved 364 case reports related to CP from 2011 to 2020, and ranked the 100 most cited cases (**Table S1**). Among the top 100 case reports, the highest number of citations was 99 (the first article) and the lowest number was 17 (the last 6 articles). Overall, the top 100 case reports were cited 1743 times, with a median of 11 times, with an average of 174.3 times per article. According to the average annual citations, the top article is cited 19.8 times per year (the first article). The bottom article articles are cited 0.8 times a year (articles 93 and 94). The top 100 case reports could provide attractive information on particular and certainly infrequent cases, such as pathological characteristics, clinical manifestations and so on, which play important roles in a more comprehensive understanding of CP. At the same time, it also shows the difference of some surgical methods, the effect of radiotherapy and the exploration of targeted therapy.

### Analysis of the CP Hotspots

**Figure 5:**
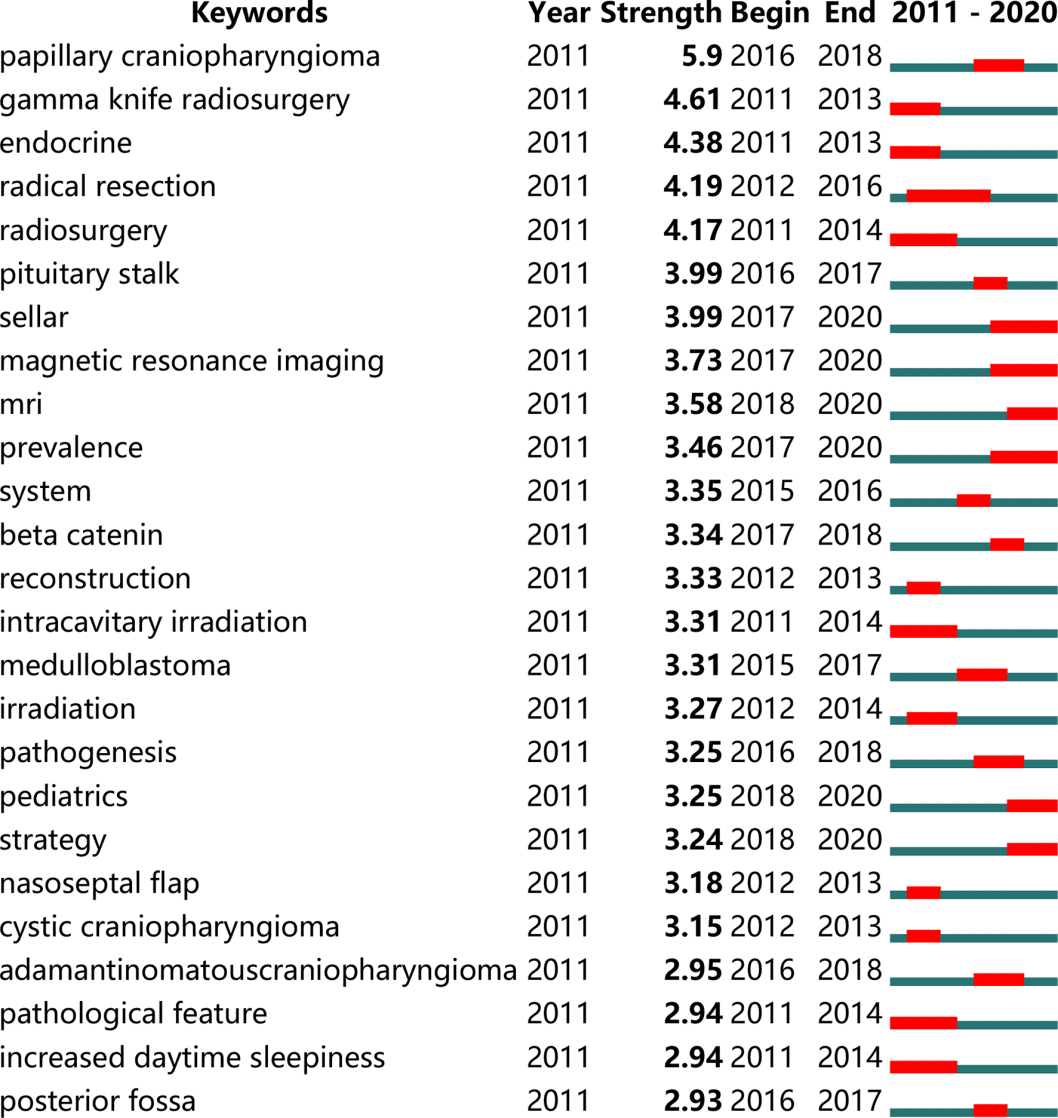
The top 25 terms with the strongest citation bursts during 2011 to 2020. Conducted by CiteSpace (version 5.7.R3, Drexel University).

**Figure 6:**
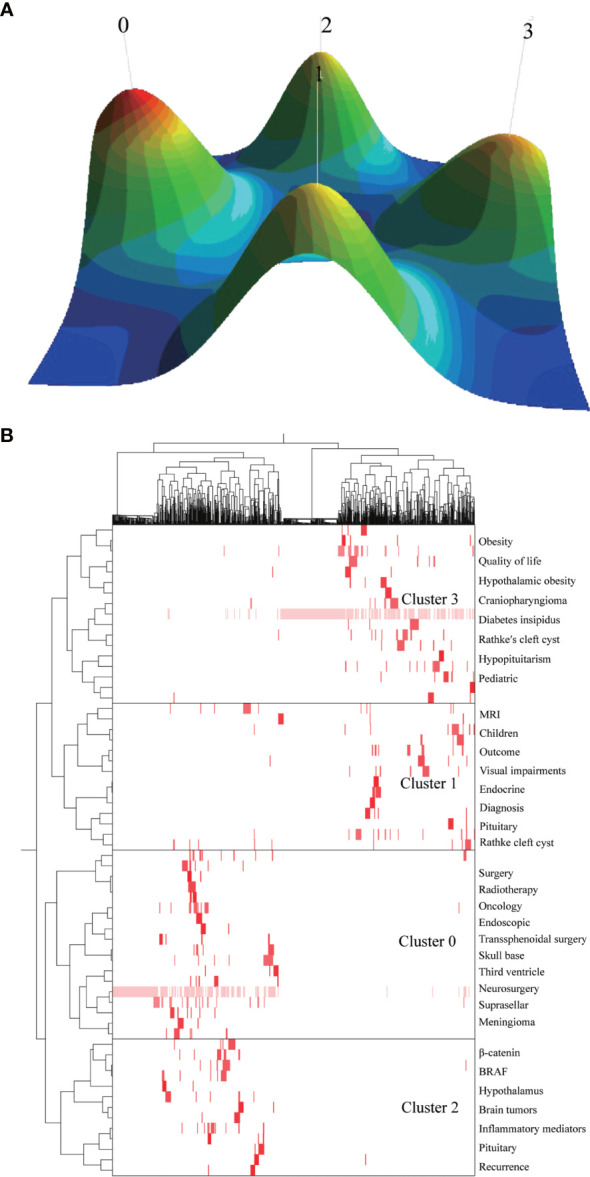
Biclustering of highly frequent major keywords and literatures on craniopharyngioma. Mountain visualization **(A)** and matrix visualization **(B)** Conducted by gCLUTO (version1.0, University of Minnesota).

We used BICOMB to extract and statistically analyze the data downloaded from the Web of science. For a more comprehensive analysis, we included keywords with a frequency greater than 10 (including 10) and generated a matrix for follow-up analysis, which accounted for 31.88% of all words. According to the 25 terms with the largest number of citations from 2011 to 2020, the time trend of hot spot transfer was analyzed (**Figure 5**). Through “gCLUTO”, we used the biclustering method to sort 4 different clusters and used mountain (**Figure 6A**) and matrix visualization (**Figure 6B**) to map the relationship between source literature and keywords. The mountain visualization can more intuitively understand the content of high-dimensional dataset. In mountain visualization, peaks 0-3 represent different clusters. Peak, volume, altitude, and color are all used to depict information about the associated category. The distance between a pair of peaks on a plane indicates the relative similarity of their categories. The altitude of each peak is positively correlated with the internal similarity of the categories. The size of the peak is proportional to the number of main keywords contained in the category. Finally, the color of the peak represents the standard deviation within the category. Red indicates a low deviation, while blue indicates a higher deviation. In matrix visualization, the column tags represent source literatures and row tags represent keywords. The cluster trees on the left represent frequent keywords associations and the cluster trees above represent literature associations. The matrix values are represented graphically, and their colors describe the frequency at which keywords appear in a literature. The color gradually deepens from white to red, indicating a gradual increase in significance. The above-mentioned high-frequency words were divided into 4 categories, and the representative literatures of each category were studied and summarized. Finally, we have identified 4 hotspots:

(0) Surgery and radiotherapy of CP
(I) Early diagnosis of CP
(II) Mechanisms/pathophysiology of CP
(III) Treatment of complications of CP

## Discussion

Through statistics and quantitative analysis, we found that the number of articles on CP published in the last 5 years from 2011 to 2020 was more than that in the previous 5 years. Although the research direction of CP was relatively extensive, there is a lack of summary and analysis of research hotspots. In this paper, we got 4 clusters through the biclustering analysis, and use the top 100 case reports to supplement the specific and uncommon information. Focusing on discussing and explaining these clusters can help identify valuable future directions.

Cluster 0 mainly highlights (0) Surgery and radiotherapy of CP. CPs are located in the sellar region. Surgical treatment may damage the optic chiasma and hypothalamus-pituitary axis, so surgeons need enough experience and good surgical skills to complete the operation. At present, there are many surgical methods, whether radical surgery or limited surgery, with an emphasis on protecting the hypothalamus and visual integrity and quality of life after treatment (9–12). When surgeons are experienced and consider the highest recurrence­free survival (13), safe gross-total resection remains the goal (14). Endoscopic transnasal and microscopic transcranial surgery have become the standard methods for the treatment of CP. However, the approach selection mode of CP is still a discussion point. The main surgical approaches for CP can be divided into five categories: anterolateral transcranial, midline transcranial, extended endoscopic transnasal, intraventricular and lateral transcranial. Each method has its advantages and limitations. The personalized surgical scheme customized for individual CP patients based on multiple factors is an important research direction to improve the prognosis in the future (15). However, surgical treatment alone, sometimes, may not be appropriate for tumors invading the hypothalamus (16). Radiotherapy alone (17) or radiotherapy combined with limited surgical treatment has become an option for some doctors. Advances in fractionated radiotherapy have made treatment more accurate, thereby reducing the volume of normal brain structures receiving high doses of radiation. Although its purpose is to reduce the toxicity caused by late radiation, this potential advantage has not been confirmed in prospective studies, and it is also a direction that needs further verification in the future (18).

Cluster 1 mainly highlights (I) Early diagnosis of CP. The diagnosis of child CP is usually made late, with nonspecific symptoms characterized by elevated intracranial pressure (such as nausea and headache). These manifestations usually occur years after the initial symptoms (19, 20). Further progress can lead to visual impairments (62–84% of patients) and endocrine deficits (52–87% of patients) (21). Sexual dysfunction caused by hypothalamic-pituitary gonadotropin deficiency and hyperprolactinemia is a special symptom in adult CP (22, 23). Complications such as visual impairment, central diabetes insipidus, hypothalamic obesity, reduced sexual function, and psychiatric alterations seriously affect the quality of life of patients. So far, studies on history before diagnosis and the prognostic relevance of duration of history and specific clinical manifestations have been limited to the pediatric age group. Improving the relevant research of other age groups plays a great role in the comprehensive understanding of CP, so it will become an indispensable focus of CP research in the future. On this basis, according to the characteristics of different age groups, formulate corresponding early diagnosis schemes to realize the early intervention of CP, and finally reduce the occurrence of complications and improve the prognosis of patients.

Cluster 2 mainly highlights (II) Mechanisms/pathophysiology of CP. CP is an epithelial tumor that occurs along the craniopharyngeal duct. In this regard, it is particularly essential to improve our understanding of the pathogenesis/pathophysiology of CP to develop targeted therapies that effectively prevent progression and hypothalamic involvement. Histopathologically, it consists of ACP and papillary PCP. ACPs are driven by somatic mutations of CTNNB1, which causes the β-catenin pathway not to be degraded effectively, accumulates in cells, and further leads to overactivation of the Wnt-β-catenin pathway (24, 25). A single cell or cell mass in which β-catenin accumulates causes the tumor to secrete too many growth factors and cytokines, activating specific pathways in surrounding nearby tumor cells (26–28). This secretory phenotype is consistent with the activation of the tumor senescence-associated secretory phenotype (SASP). And some studies have proved that SASP plays a vital role in the pathogenesis of ACP (26, 29, 30). Inflammatory mediators are also the key points of CP. Many inflammatory mediators, chemokines, and cytokines are expressed in the cystic and solid components of human ACP (31–34). These molecules may facilitate the escape of immune surveillance. By contrast, only somatic BRAFV600E mutations have been found in PCP (35, 36). Through MAPK activation, the mutation can transform normal SOX2+ stem cells in the pituitary into PCP tumor initiation cells (37). The potential role of cell senescence in PCP has not been determined, nor has the expression of inflammatory mediators in PCP been investigated. However, some studies (38, 39) have shown that the influence of inflammation and cell senescence on CP has become a focus and hotspot in this field, and there are still huge deficiencies in these two aspects. In the future, researchers engaged in CP related research are still needed to further explore these two aspects, so as to achieve effective treatment to prevent CP progression and hypothalamic involvement. At the same time, targeted therapy for CP specific mutation points (CTNNB1 and BRAF) is also an exciting research direction. Many of the top 100 cited case reports are about this aspect (40–43).

Cluster 3 mainly highlights (III) Treatment of complications of CP. No matter what kind of treatment for CP patients, the prevention of long-term morbidity should be a major strategic consideration. The functional and social independence of patients is the goal of treatment (39). With the development of medical technology, the rational use of corticosteroids and antibiotics to reduce inflammation and infection, the perioperative morbidity and mortality of CP patients have been greatly improved. Moreover, most of the complications of CP and its treatment can be managed through drugs and psychological, psychiatric and emotional care (44, 45). In recent years, our understanding of the neuropsychological adverse effects of CP has been dramatically improved. However, the treatment effect is not available for hypothalamic syndrome and its main clinical manifestations (obesity and neuropsychological defects). In order to improve the therapeutic effect, we must further understand how CP causes hypothalamic syndrome and its main clinical manifestations, and further explore the pathogenesis of these complications. In short, we need to keep exploring in the future to avoid these complications as much as possible and get effective treatment when symptoms appear.

Although we have analyzed the literatures on CP from 2011 to 2020 as comprehensively as possible, and analyzed top 100 cited case reports in the past 10 years as a supplement, there are still some limitations. Some literatures [such as Craniopharyngioma (46), Q1, IF=52.322] published recently, through the analysis of citation information, cannot well highlight its importance. Simultaneously, the database is still constantly updated, and there may be differences between our bibliometrics analysis and the actual publishing conditions. For any bibliometric analysis of CP, it should also be pointed out that there is a limitation, that is, only positive and excellent results are usually published in the most prestigious journals, so true, non-excellent results obtained in most patients still hidden in scientific analysis. Most of the published CPs surgery series are just the final work of a very prestigious surgeon/clinician working in a specialized center for decades. This process limits the opportunity to mix the treatment ideas/results of different institutions/authors for collaborative or collaborative research.

## Conclusions

We summarized the literatures related to CP from 2011 to 2020, including source countries and institutions, authors, published journals, etc. Then, based on these publications, we analyze research hotspots and predict future trends. Reviewing the previous studies on CP, we discussed the four aspects of CP and found the key research. Subsequently, we included the top 100 cited case reports in the analysis to enrich our discussion. We fully and specifically discussed the shortcomings of these priorities and the importance of achieving breakthroughs in these priorities for the treatment of CP patients. We believe that our research will help to determine the valuable future direction for CP research, and the mentioned hot spots will make major breakthroughs in the future.

## Data Availability Statement

The original contributions presented in the study are included in the article/**Supplementary Material**. Further inquiries can be directed to the corresponding authors.

## Author Contributions 

YL led the team and was responsible for all aspects of the project. TL and AY substantially contributed to the methods, data acquisition, results, and interpretation. SZ, YC, and BN participated in designing and writing the manuscript. GL revised this manuscript critically for important intellectual content. JF gave final approval of the manuscript. All authors contributed to the article and approved the submitted version.

## Funding

This work was supported by grants from the Science and technology projects of Guangdong Province (2017A020215191).

## Conflict of Interest

The authors declare that the research was conducted in the absence of any commercial or financial relationships that could be construed as a potential conflict of interest.

## Publisher’s Note

All claims expressed in this article are solely those of the authors and do not necessarily represent those of their affiliated organizations, or those of the publisher, the editors and the reviewers. Any product that may be evaluated in this article, or claim that may be made by its manufacturer, is not guaranteed or endorsed by the publisher.

## Supplementary Material

Supplementary Table 1 Top 100 cited case reports during 2011-2020.
