# Supplementary material for: Status Quo and Research Trends of Craniopharyngioma Research: A 10-Year Bibliometric Analyses (From 2011 to 2020)
Source: Front Oncol. 2021 Sep 30;11:744308. doi: 10.3389/fonc.2021.744308 (PMC8516404; doi:10.3389/fonc.2021.744308)
Supplement: Supplementary Table 1 — Top 100 cited case reports during 2011-2020. [file Table_1.docx]

| **Table S1** Top 100 cited case reports during 2011-2020 | | | |  |  |  |
| --- | --- | --- | --- | --- | --- | --- |
| WOS Citation Rank | Investigator | Year | Title | Total Citations | Average annual Citations | Rank of Average annual Citations |
| 1 | Brastianos, PK et al. | 2016 | Dramatic Response of BRAF V600E Mutant Papillary Craniopharyngioma to Targeted Therapy | 99 | 19.80 | 1 |
| 2 | Clark, AJ et al. | 2013 | A systematic review of the results of surgery and radiotherapy on tumor control for pediatric craniopharyngioma | 59 | 7.38 | 5 |
| 3 | Zoicas, F et al. | 2013 | GLP-1 analogues as a new treatment option for hypothalamic obesity in adults: report of nine cases | 57 | 7.13 | 6 |
| 4 | Prieto, R et al. | 2013 | Predictive Factors for Craniopharyngioma Recurrence: A Systematic Review and Illustrative Case Report of a Rapid Recurrence | 55 | 6.88 | 7 |
| 5 | Liu,JK et al. | 2011 | Surgical nuances for removal of retrochiasmatic craniopharyngioma via the endoscopic endonasal extended transsphenoidal transplanum transtuberculum approach | 48 | 4.80 | 19 |
| 6 | Aylwin, SJB et al. | 2016 | Pronounced response of papillary craniopharyngioma to treatment with vemurafenib, a BRAF inhibitor | 46 | 9.20 | 3 |
| 7 | Borges, MT et al. | 2011 | Spindle cell oncocytoma with late recurrence and unique neuroimaging characteristics due to recurrent subclinical intratumoral bleeding | 39 | 3.90 | 21 |
| 8 | Aslan, IR et al. | 2011 | Bariatric surgery in a patient with complete MC4R deficiency | 37 | 3.70 | 24 |
| 9 | Conger, AR et al. | 2014 | Endoscopic extended transsphenoidal resection of craniopharyngiomas: nuances of neurosurgical technique | 35 | 5.00 | 17 |
| 10 | Fleming, AJ et al. | 2012 | Brain Tumors in Children | 35 | 3.89 | 22 |
| 11 | Ren, XH et al. | 2012 | Clinical, radiological, and pathological features of 24 atypical intracranial epidermoid cysts | 34 | 3.78 | 23 |
| 12 | Fujisawa, H et al. | 2012 | Spindle Cell Oncocytoma of the Adenohypophysis With Marked Hypervascularity -Case Report- | 32 | 3.56 | 26 |
| 13 | Morisako, H et al. | 2016 | Aggressive surgery based on an anatomical subclassification of craniopharyngiomas | 31 | 6.20 | 9 |
| 14 | Himes, BT | 2019 | Recurrent papillary craniopharyngioma with BRAF V600E mutation treated with dabrafenib: case report | 29 | 14.50 | 2 |
| 15 | Hess, CB et al. | 2016 | Exposure Risks Among Children Undergoing Radiation Therapy: Considerations in the Era of Image Guided Radiation Therapy | 29 | 5.80 | 12 |
| 16 | Wang, L et al. | 2016 | Bypass surgery to treat symptomatic fusiform dilation of the internal carotid artery following craniopharyngioma resection: report of 2 cases | 28 | 5.60 | 13 |
| 17 | Nikolaev, A et al. | 2016 | Association between Hyperprolactinemia and Granulomatous Mastitis | 28 | 5.60 | 13 |
| 18 | Liu, JK et al. | 2016 | Microscopic versus endoscopic approaches for craniopharyngiomas: choosing the optimal surgical corridor for maximizing extent of resection and complication avoidance using a personalized, tailored approach | 27 | 5.40 | 16 |
| 19 | Hsu, EA et al | 2018 | Oxytocin and Naltrexone Successfully Treat Hypothalamic Obesity in a Boy Post-Craniopharyngioma Resection | 26 | 8.67 | 4 |
| 20 | Rostami, E et al. | 2017 | Recurrent papillary craniopharyngioma with BRAFV600E mutation treated with neoadjuvant-targeted therapy | 26 | 6.50 | 8 |
| 21 | Xie, T et al. | 2011 | 3D-FIESTA MR images are useful in the evaluation of the endoscopic expanded endonasal approach for midline skull-base lesions | 26 | 2.60 | 35 |
| 22 | Rahmani, R et al. | 2015 | Parasellar xanthogranulomas | 25 | 4.17 | 20 |
| 23 | Sofela, AA et al. | 2014 | Malignant Transformation in Craniopharyngiomas | 24 | 3.43 | 28 |
| 24 | Kamoshima, Y et al. | 2011 | Xanthogranuloma of the Sellar Region of Children: Series of Five Cases and Literature Review | 24 | 2.40 | 39 |
| 25 | Ogilvy, CS et al. | 2011 | Stent-Assisted Coiling Treatment of Pediatric Traumatic Pseudoaneurysm Resulting from Tumor Surgery | 24 | 2.40 | 39 |
| 26 | Kopelovich, JC et al. | 2012 | Pneumocephalus with BiPAP use after transsphenoidal surgery | 23 | 2.56 | 36 |
| 27 | Filho, LFSD et al. | 2014 | Supraorbital Eyebrow Craniotomy for Removal of Intraaxial Frontal Brain Tumors: A Technical Note | 22 | 3.14 | 29 |
| 28 | Jinguji, S et al. | 2013 | Endoscopic biopsies of lesions associated with a thickened pituitary stalk | 20 | 2.50 | 37 |
| 29 | Finzi, G et al. | 2014 | Mixed pituitary adenoma/craniopharyngioma: clinical, morphological, immunohistochemical and ultrastructural study of a case, review of the literature, and pathogenetic and nosological considerations | 19 | 2.71 | 32 |
| 30 | Gao, SZ et al. | 2011 | Malignant transformation of craniopharyngioma: case report and review of the literature | 18 | 1.80 | 52 |
| 31 | Gatta, B et al. | 2013 | Is bariatric surgery really inefficient in hypothalamic obesity? | 17 | 2.13 | 48 |
| 32 | Gu, Y et al. | 2015 | Suprachiasmatic translamina terminalis corridor used in endoscopic endonasal approach for resecting third ventricular craniopharyngioma | 16 | 2.67 | 33 |
| 33 | van Santen, HM et al. | 2015 | Effects of T3 treatment on brown adipose tissue and energy expenditure in a patient with craniopharyngioma and hypothalamic obesity | 16 | 2.67 | 33 |
| 34 | Jin, GS et al. | 2013 | Collision tumors of the sella: coexistence of pituitary adenoma and craniopharyngioma in the sellar region | 16 | 2.00 | 49 |
| 35 | Miyajima, Y et al. | 2011 | Rathke's Cleft Cyst With Xanthogranulomatous Change-Case Report | 16 | 1.60 | 59 |
| 36 | Hammond, JL et al. | 2011 | Functional analysis and treatment of aggressive behavior following resection of a craniopharyngioma | 16 | 1.60 | 59 |
| 37 | Nishioka, H et al. | 2016 | Endoscopic Endonasal Surgery for Purely Intrathird Ventricle Craniopharyngioma | 15 | 3.00 | 30 |
| 38 | Simmons, JH et al. | 2012 | Treatment with Glucagon-Like Peptide-1 Agonist Exendin-4 in a Patient with Hypothalamic Obesity Secondary to Intracranial Tumor | 15 | 1.67 | 56 |
| 39 | Lauriola, L et al. | 2011 | De novo malignant craniopharyngioma: case report and literature review | 15 | 1.50 | 66 |
| 40 | Keswani, A et al. | 2012 | Lymphomatosis cerebri presenting with orthostatic hypotension, anorexia, and paraparesis | 14 | 1.56 | 65 |
| 41 | Lee, HS et al. | 2011 | Moyamoya Syndrome Precipitated by Cranial Irradiation for Craniopharyngioma in Children | 14 | 1.40 | 71 |
| 42 | Wang, W et al. | 2015 | Malignant transformation of craniopharyngioma with detailed follow-up | 13 | 2.17 | 47 |
| 43 | Pulickal, GG et al. | 2014 | Tension pneumocephalus | 13 | 1.86 | 51 |
| 44 | Juratli, TA et al. | 2019 | Targeted treatment of papillary craniopharyngiomas harboring BRAF V600E mutations | 12 | 6.00 | 10 |
| 45 | Mattogno, PP et al. | 2019 | First Case of Primary Sellar/Suprasellar-Intraventricular Ewing Sarcoma: Case Report and Review of the Literature | 12 | 6.00 | 10 |
| 46 | Cook, N et al. | 2016 | Parent observed neuro-behavioral and pro-social improvements with oxytocin following surgical resection of craniopharyngioma | 12 | 2.40 | 39 |
| 47 | Mohanty, A et al. | 2013 | Initial Experience with Endoscopic Side Cutting Aspiration System in Pure Neuroendoscopic Excision of Large Intraventricular Tumors | 12 | 1.50 | 66 |
| 48 | Mortini, P et al. | 2013 | The Combined Interhemispheric Subcommissural Translaminaterminalis Approach for Large Craniopharyngiomas | 12 | 1.50 | 66 |
| 49 | Page-Wilson, G et al. | 2012 | Hypothalamic obesity in patients with craniopharyngioma: treatment approaches and the emerging role of gastric bypass surgery | 12 | 1.33 | 73 |
| 50 | Borrill, R et al. | 2019 | Papillary craniopharyngioma in a 4-year-old girl with BRAF V600E mutation: a case report and review of the literature | 11 | 5.50 | 15 |
| 51 | Capatina, C et al. | 2018 | CRANIOPHARYNGIOMA - CLINICAL AND THERAPEUTIC OUTCOME DATA IN A MIXED COHORT OF ADULT AND PAEDIATRIC CASES | 11 | 3.67 | 25 |
| 52 | Bal, E et al. | 2016 | Endoscopic Endonasal Transsphenoidal Surgery, A Reliable Method for Treating Primary and Recurrent/Residual Craniopharyngiomas: Nine Years of Experience | 11 | 2.20 | 46 |
| 53 | Iacoangeli, M et al. | 2014 | A Rare Case of Chordoma and Craniopharyngioma Treated by an Endoscopic Endonasal, Transtubercular Transclival Approach | 11 | 1.57 | 64 |
| 54 | Yeung, JT et al. | 2012 | Pegylated interferon-alpha-2b for children with recurrent craniopharyngioma Clinical article | 11 | 1.22 | 79 |
| 55 | Kordes, U et al. | 2011 | Ectopic Craniopharyngioma | 11 | 1.10 | 86 |
| 56 | Panesar, SS et al. | 2019 | Patient-specific 3-dimensionally printed models for neurosurgical planning and education | 10 | 5.00 | 17 |
| 57 | Zhao, Y et al. | 2017 | Collision tumors composed of meningioma and growth hormone-secreting pituitary adenoma in the sellar region Case reports and a literature review | 10 | 2.50 | 37 |
| 58 | Bailey, S et al. | 2015 | Intracystic interferon therapy in childhood craniopharyngioma: who, when and how? | 10 | 1.67 | 56 |
| 59 | Tosaka, M et al. | 2015 | Superficial Siderosis of the Central Nervous System Caused by Hemorrhagic Intraventricular Craniopharyngioma: Case Report and Literature Review | 10 | 1.67 | 56 |
| 60 | Mascelli, S et al. | 2012 | Analysis of NADP+-dependent isocitrate dehydrogenase-1/2 gene mutations in pediatric brain tumors: report of a secondary anaplastic astrocytoma carrying the IDH1 mutation | 10 | 1.11 | 83 |
| 61 | Jung, TY et al. | 2012 | Operative outcomes and adjuvant treatment of purely third ventricle craniopharyngioma after a transcallosal approach | 10 | 1.11 | 83 |
| 62 | Okada, T et al. | 2012 | Coexistence of adamantinomatous and squamous-papillary type craniopharyngioma: Case report and discussion of etiology and pathology | 10 | 1.11 | 83 |
| 63 | Pekic, S et al. | 2011 | Case seminar: a young female with acute hyponatremia and a sellar mass | 10 | 1.00 | 87 |
| 64 | Schlatter, SM et al. | 2018 | Rathke's Cleft Cyst as Origin of a Pediatric Papillary Craniopharyngioma | 9 | 3.00 | 30 |
| 65 | Bhogal, P et al. | 2017 | The use of a stent-retriever to cause mechanical dilatation of a vasospasm secondary to iatrogenic subarachnoid haemorrhage | 9 | 2.25 | 43 |
| 66 | Hernandez-Estrada, RA et al. | 2017 | Cholesterol granulomas presenting as sellar masses: a similar, but clinically distinct entity from craniopharyngioma and Rathke's cleft cyst | 9 | 2.25 | 43 |
| 67 | Liu, HL et al. | 2017 | Sellar and Suprasellar Granular Cell Tumor of Neurohypophysis | 9 | 2.25 | 43 |
| 68 | Takano, S et al. | 2015 | Neuroendoscopy Followed by Radiotherapy in Cystic Craniopharyngiomas-a Long-Term Follow-Up | 9 | 1.50 | 66 |
| 69 | Meijneke, RWH et al. | 2015 | Hypothalamic obesity after treatment for craniopharyngioma: the importance of the home environment | 9 | 1.50 | 66 |
| 70 | Mori, R et al. | 2014 | Parallel Insertion Endoscopic Technique for Precise Catheter Placement in Cystic Craniopharyngiomas | 9 | 1.29 | 76 |
| 71 | Kostadinov, S et al. | 2014 | Fetal Craniopharyngioma: Management, Postmortem Diagnosis, and Literature Review of an Intracranial Tumor Detected in Utero | 9 | 1.29 | 76 |
| 72 | Miljic, D et al. | 2014 | Adipsic diabetes insipidus and venous thromboembolism (VTE): recommendations for addressing its hypercoagulability | 9 | 1.29 | 76 |
| 73 | Sakuta, K et al. | 2012 | Possible Mechanism of Secondary Narcolepsy with a Long Sleep Time Following Surgery for Craniopharyngioma | 9 | 1.00 | 87 |
| 74 | Vogel, TD et al. | 2011 | Tumor bleeding from a de novo aneurysm associated with optic glioma Case report | 9 | 0.90 | 91 |
| 75 | Elfving, M et al. | 2011 | Ectopic recurrence of a craniopharyngioma in a 15-year-old girl 9 years after surgery and conventional radiotherapy: case report | 9 | 0.90 | 91 |
| 76 | Garcia-Navarro, V et al. | 2011 | Use of a side-cutting aspiration device for resection of tumors during endoscopic endonasal approaches | 9 | 0.90 | 91 |
| 77 | Cramer, SD et al. | 2011 | Sellar xanthogranuloma in a dog | 9 | 0.90 | 91 |
| 78 | Gabel, BC et al. | 2017 | Unusual and Rare Locations for Craniopharyngiomas: Clinical Significance and Review of the Literature | 8 | 2.00 | 49 |
| 79 | Gilliland, T et al. | 2016 | Resolution of non-alcoholic steatohepatitis after growth hormone replacement in a pediatric liver transplant patient with panhypopituitarism | 8 | 1.60 | 59 |
| 80 | Bretault, M et al. | 2016 | Postprandial GLP-1 Secretion After Bariatric Surgery in Three Cases of Severe Obesity Related to Craniopharyngiomas | 8 | 1.60 | 59 |
| 81 | Cuesta, M et al. | 2016 | Heterogenous patterns of recovery of thirst in adult patients with adipsic diabetes insipidus | 8 | 1.60 | 59 |
| 82 | Ricarte, IF et al. | 2015 | Symptomatic Cerebral Vasospasm and Delayed Cerebral Ischemia Following Transsphenoidal Resection of a Craniopharyngioma | 8 | 1.33 | 73 |
| 83 | Alomari, AK et al. | 2015 | Craniopharyngioma arising in a Rathke's cleft cyst: case report | 8 | 1.33 | 73 |
| 84 | Ogawa, Y et al. | 2014 | Spontaneous Alteration from Rathke's Cleft Cyst to Craniopharyngioma-Possible Involvement of Transformation Between These Pathologies | 8 | 1.14 | 80 |
| 85 | Tawk, RG et al. | 2014 | Transciliary Supraorbital Approach (Eyebrow Approach) for Resection of Retrochiasmatic Craniopharyngiomas: An Alternative Approach, Case Series, and Literature Review | 8 | 1.14 | 80 |
| 86 | Beaty, NB et al. | 2014 | Adamantinomatous Craniopharyngioma Containing Teeth | 8 | 1.14 | 80 |
| 87 | Xie, T et al. | 2013 | Suprasellar Hemangioblastoma Mimicking a Craniopharyngioma: Result of Extended Endoscopic Transsphenoidal Approach-Case Report | 8 | 1.00 | 87 |
| 88 | Demetriades, AK et al. | 2013 | The ventriculocholecystic shunt: two case reports and a review of the literature | 8 | 1.00 | 87 |
| 89 | Khalatbari, MR et al. | 2012 | Isolated primary craniopharyngioma in the cerebellopontine angle | 8 | 0.89 | 95 |
| 90 | Dunham, C et al. | 2012 | Infant brain tumors: a neuropathologic population-based institutional reappraisal | 8 | 0.89 | 95 |
| 91 | Choudhry, OJ et al. | 2012 | Giant Suprasellar Rathke's Cleft Cyst Mimicking Craniopharyngioma: Implications for a Spectrum of Cystic Epithelial Lesions of Ectodermal Origin | 8 | 0.89 | 95 |
| 92 | Jakobs, M et al. | 2012 | Ectopic recurrent craniopharyngioma of the frontal bone Case report | 8 | 0.89 | 95 |
| 93 | Park, YS et al. | 2011 | Recurrence rates after neuroendoscopic fenestration and Gamma Knife surgery in comparison with subtotal resection and Gamma Knife surgery for the treatment of cystic craniopharyngiomas | 8 | 0.80 | 99 |
| 94 | Bozbuga, M et al. | 2011 | Primary cerebellopontine angle craniopharyngioma in a patient with Gardner syndrome | 8 | 0.80 | 99 |
| 95 | Grob, S et al. | 2019 | Targeting IL-6 Is a Potential Treatment for Primary Cystic Craniopharyngioma | 7 | 3.50 | 27 |
| 96 | Hanaoka, Y et al. | 2018 | Calcified ectopic TSH-secreting pituitary adenoma mimicking craniopharyngioma: a rare case report and literature review | 7 | 2.33 | 42 |
| 97 | Ciappetta, Pet al. | 2017 | Anatomic Dissection of Arachnoid Membranes Encircling the Pituitary Stalk on Fresh, Non-Formalin-Fixed Specimens: Anatomoradiologic Correlations and Clinical Applications in Craniopharyngioma Surgery | 7 | 1.75 | 53 |
| 98 | Castro-Dufourny, I et al. | 2017 | Hypothalamic obesity after craniopharyngioma surgery: Treatment with a long acting glucagon like peptide 1 derivated | 7 | 1.75 | 53 |
| 99 | Jeong, TS et al. | 2017 | Malignant Transformation of Craniopharyngioma without Radiation Therapy: Case Report and Review of the Literature | 7 | 1.75 | 53 |
| 100 | Nash, R et al. | 2016 | Management strategy for treatment of vasospasm following transsphenoidal excision of craniopharyngioma | 7 | 1.40 | 71 |
